# *From concept to proof:* developing a neurofeedback-fNIRS protocol to train self-regulation for music performance anxiety in adolescent musicians

**DOI:** 10.3389/fpsyg.2026.1746761

**Published:** 2026-04-29

**Authors:** Nery Borges, Pedro Rodrigues Ribeiro, Sofia Serra, Lurdes Veríssimo, Pedro Dias, Nádia Moura, Inês Silva, Isaac Raimundo, Patrícia Oliveira-Silva

**Affiliations:** 1 University of Aveiro, Institute of Ethnomusicology – Centre for Studies in Music and Dance (INET-md), Department of Communication and Arts, Aveiro, Portugal; 2 Universidade Católica Portuguesa, Human Neurobehavioral Laboratory (HNL), Porto, Portugal; 3 Universidade Católica Portuguesa, Faculty of Education and Psychology (FEP), Porto, Portugal; 4 Universidade Católica Portuguesa, Research Centre for Human Development (CEDH), Porto, Portugal; 5 University of the Azores, Department of Psychology, Ponta Delgada, Portugal; 6 University of Coimbra, CEIS20 – Centre for Interdisciplinary Studies, Faculty of Arts and Humanities, Coimbra, Portugal; 7 NOVA University of Lisbon, INET-md, Lisbon, Portugal

**Keywords:** adolescents, gradual exposure, music performance anxiety, neurofeedback-fNIRS, neurophysiological self-regulation

## Abstract

**Introduction:**

Music Performance Anxiety (MPA) is a prevalent phenomenon among young musicians, affecting both well-being and artistic performance. Despite its early onset and impact, effective interventions supporting the development of self-regulation of MPA in adolescents remain limited, particularly in relation to neurofeedback-based approaches. In response, this study investigates the feasibility of a Neurofeedback–Functional Near-Infrared Spectroscopy (NFDK-fNIRS) training protocol designed to support this process within a proof-of-concept research framework.

**Methods:**

The protocol alternated MPA induction and regulation within audio-guided sessions conducted in school settings to ensure ecological validity and to integrate top-down control with gradual exposure to performance contexts. The conceptual development and operationalization of the protocol resulted in a theoretical–methodological framework combining gradual exposure, guided imagery, and neurophysiological self-regulation training. This protocol was implemented through standardized auditory scripts and systematic procedures to ensure replicability and experimental coherence. The proof-of-concept implementation was conducted with three female adolescent participants (aged 12–13) across four individual sessions designed to progressively develop physiological self-regulation in response to MPA.

**Results:**

The procedure demonstrated technical feasibility, hemodynamic signal stability, and participants’ gradual adaptation to the training. fNIRS data revealed increased modulation of the dorsolateral prefrontal cortex, consistent with top-down self-regulatory learning mechanisms. Researchers’ reflective journals emphasized practical aspects such as equipment calibration, session duration, and participant engagement.

**Discussion:**

Altogether, the findings indicate that NFDK-fNIRS represents a technically feasible and ecologically applicable approach for training self-regulation of MPA in adolescents, providing a solid foundation for future studies involving larger samples and controlled validation of the protocol.

## 1 Introduction

Music Performance Anxiety (MPA) is a persistent and context-specific form of anxiety experienced in musical performance settings and is recognized as one of the most prevalent psychological stressors among musicians (Kenny, 2011). MPA levels tend to intensify in evaluative situations, such as competitions, auditions, and public performances, where heightened scrutiny can directly affect artistic development and career progression (LeBlanc, 1994; Papageorgi et al., 2007; Borges et al., 2023). At moderate levels, MPA-related arousal may enhance focus, motivation, and an optimal state that facilitates performance. However, when arousal exceeds the functional activation threshold, becoming physiologically excessive or cognitively disruptive, it shifts toward maladaptively, impairing wellbeing and performance quality (Papageorgi et al., 2007, 2013; Papageorgi, 2021). This distinction aligns with the Yerkes and Dodson (1908) law, which posits that performance efficiency follows an inverted-U relationship with arousal, being optimal at moderate levels and declining when it is either too low or too high.

Understanding the scope and persistence of MPA is therefore crucial for contextualizing these gaps. MPA exerts a profound influence on musicians’ subjective experience and performance quality. In their systematic review, Fernholz et al. (2019) reported that the prevalence of MPA can reach up to 60% among professional musicians and music students. Similarly, Barros et al. (2022) found that among university-level music students, this rate may reach as high as 83.1%. In young music students aged between 12 and 19, 11% exhibited elevated levels of MPA, with the highest incidence occurring between the ages of 14 and 19 (Papageorgi, 2022). Cumulative evidence further suggests a progressive increase in anxiety throughout formal musical training, reinforcing the importance of early preventive and regulatory strategies (Patston and Osborne, 2016; Papageorgi, 2021, 2022; Wang and Yang, 2024). Given the early emergence of MPA and its progression throughout musical training, adolescence represents a particularly relevant developmental stage for preventive and self-regulatory interventions.

A variety of therapeutic approaches have been applied in the treatment of MPA, including cognitive behavioural therapy (Osborne et al., 2007; Kenny and Halls, 2018), acceptance and commitment therapy (Osborne et al., 2007; Clarke et al., 2020; Mahony et al., 2022), music therapy (Kim, 2005, 2008; Clements-Cortés et al., 2024), yoga and mindfulness-based interventions (Stern et al., 2012; Khalsa et al., 2013; Butzer et al., 2016), virtual reality exposure (Williamon et al., 2014; Bissonnette et al., 2016; Aufegger et al., 2017; Choi et al., 2024), biofeedback and physiological techniques (Thurber et al., 2010; Wells et al., 2012; Barros et al., 2019), hypnotherapy (Brooker, 2018), live stage exposure (Candia et al., 2023), and multimodal strategies (Braden et al., 2015; Cohen and Bodner, 2019; Moral-Bofill et al., 2022; Gómez-López and Sánchez-Cabrero, 2024).

Despite the diversity of approaches identified, systematic reviews indicate that the current body of evidence remains limited, particularly among adolescents, characterized by small sample sizes, methodological heterogeneity, and a lack of randomized controlled trials (Burin and Osório, 2016; Gómez-López and Sánchez-Cabrero, 2023; Lima et al., 2024; Kinney et al., 2025; Nicholl and Abbott, 2025). To date, these reviews have not identified studies applying neurofeedback (NFDK) as an intervention strategy for MPA. While preliminary work (e.g., Pereira, 2024) has begun to explore NFDK through structured training sessions targeting subjective MPA and emotional well-being, this line of research remains at an early stage. These gaps support the need for exploratory studies focusing on the feasibility and methodological coherence of emerging NFDK approaches in this domain. In this context, NFDK approaches have been proposed as promising tools for training emotional self-regulation through real-time monitoring of brain activity.

NFDK-fNIRS is an emerging neuromodulation technique that combines real-time feedback of brain activity with portable optical technology. It uses near-infrared light to measure cortical hemodynamic variations, similarly to functional magnetic resonance imaging (fMRI) (Chincarini et al., 2020). This is particularly relevant as it allows inference of cortical activity at the voxel level through neurovascular coupling, providing indirect but spatially precise information about localised brain activation patterns (Pinti et al., 2020; Yücel et al., 2021). Its non-invasive nature, low cost, and reduced susceptibility to motion artefacts enable its application in naturalistic performance contexts, offering higher ecological validity compared with fMRI, electroencephalography (EEG), and positron emission tomography (PET) (Tuscan et al., 2013; Glassman et al., 2016; Klein et al., 2024; Fagerland et al., 2025). In the specific case of NFDK-fNIRS, studies report good acceptability, safety, and ecological feasibility (Fishburn et al., 2014; Fagerland et al., 2025).

Studies have shown that prefrontal cortex activity can be modulated through real-time NFDK, supporting emotional regulation (Kohl et al., 2020; Li et al., 2025). NFDK-fNIRS applied to the dorsolateral and ventrolateral prefrontal cortices (dlPFC and vlPFC) appears particularly favorable, as it is safe and well-tolerated in both school and laboratory environments (Fishburn et al., 2014). It is sensitive to cognitive control processes, with evidence of transfer effects to functional connectivity (Schumacher et al., 2019; Yang et al., 2024; Zeng et al., 2025), and has demonstrated efficacy even in short training programmes, with measurable effects observed after only a few sessions (Agbangla et al., 2022; Godet et al., 2023).

In adolescents, NFDK-fNIRS shows encouraging potential, as this developmental period is marked by heightened neural plasticity, progressive maturation of the prefrontal cortex, and increased responsiveness to self-regulatory interventions (Baker et al., 2025; Kanwal et al., 2016). This plasticity, particularly within regions implicated in emotional regulation, such as the dlPFC and vlPFC, has been associated with greater adolescent sensitivity to NFDK training, with initial evidence of efficacy in protocols targeting these areas (Kadosh et al., 2013; Kanwal et al., 2016; Quevedo et al., 2019; Lipp and Cohen Kadosh, 2020; Ahrweiler et al., 2022; Liu et al., 2022; Baker et al., 2025). Furthermore, evidence suggests that, during adolescence, neural signatures of anxiety and depression converge within the medial prefrontal cortex, reinforcing the relevance of self-regulatory interventions at this developmental stage (Papasideris et al., 2021).

NFDK protocols display considerable heterogeneity in terms of the number of sessions, training duration, target regions, and feedback modalities, which complicates the standardization and comparison of results (Kohl et al., 2020). Evidence from EEG- and fMRI-based NFDK studies suggests that measurable effects can emerge under both short- and long-term training protocols (Kohl et al., 2020; Agbangla et al., 2022; Godet et al., 2023). The prefrontal cortex has been the principal target of training, given its central role in emotional regulation, executive control, and functional connectivity with limbic and attentional systems, particularly the amygdala and anterior cingulate cortex (Schumacher et al., 2019; Yang et al., 2024; Zeng et al., 2025). Recently, efforts toward standardization have gained momentum, with the development of manualized NFDK protocols applied in psychotherapeutic contexts, underscoring the need for greater methodological rigor and replicability (Schmidt et al., 2024). Considering the developmental sensitivity of adolescence and the potential of NFDK to support emotional self-regulation, exploring the feasibility of NFDK-fNIRS in the context of MPA represents a relevant next step.

Despite significant advances in NFDK research, no fNIRS-based studies have yet investigated the application of NFDK specifically targeting MPA in adolescents within educational contexts. This gap is particularly relevant given the impact of MPA on the wellbeing and musical development of young performers, as well as the potential of fNIRS to provide safe, ecologically valid, and adaptable self-regulation training environments. Accordingly, the present study adopted a proof-of-concept framework to develop, implement, and preliminarily examine an NFDK-fNIRS protocol designed to support self-regulation of MPA in adolescent musicians within school settings.

More specifically, the study addressed the following research questions:

(1) How can a NFDK-fNIRS protocol for MPA be conceptually developed and operationalised in a scientifically coherent and practically implementable way for adolescent musicians?
(2) Is the protocol technically and procedurally feasible when implemented in real educational settings?
(3) Do the qualitative and neurophysiological observations obtained during implementation provide preliminary indications that the protocol is functionally coherent with the training of self-regulation in MPA?

## 2 Methodology

### 2.1 Study design

This study followed a developmental methodological design with a proof-of-concept framework. It comprised two sequential components: (a) the conceptual development and operationalisation of the NFDK-fNIRS protocol for MPA, and (b) its preliminary implementation in an educational context to examine feasibility and functional coherence. This approach aligns with frameworks for the development of complex interventions, which emphasise conceptual grounding, procedural specification, and early-stage feasibility testing prior to controlled evaluation (Eldredge et al., 2016; Skivington et al., 2021).

### 2.2 Protocol conception

The protocol conception was guided by key questions that structured the definition of objectives, strategies, and methods, supporting transparency and reproducibility in the development process (French et al., 2012; Michie et al., 2017; Duncan et al., 2020). Table 1 summarises the conceptual framework underpinning the protocol design.

**Table 1:** Conceptual framework guiding the design of the NFDK-fNIRS training protocol.

Conceptual framework for NFDK-fNIRS protocol conception		
Guiding question	Purpose	Rationale/theoretical basis
How much to train?	To define a training load that maximizes self-regulation learning while ensuring feasibility and replicability in educational contexts.	Duration and frequency were based on reduced biofeedback protocols (Chaló et al., 2017) adapted to MPA (Barros et al., 2019), on evidence of cortical modulation learning in short NFDK-fNIRS and NFDK-EEG protocols (Kober et al., 2014; Kohl et al., 2020), and recommendations for school-compatible interventions involving brief, progressive sessions (Marx et al., 2015; Trambaiolli and Falk, 2023).
How to conduct the training?	To define a standardized, ecologically valid delivery method adapted to adolescents.	Structured audio guides ensured standardization, temporal control, and instructor independence, supported by evidence for auditory instruction efficacy in emotion regulation and guided imagery (Shin et al., 1999; Misaki et al., 2018; Hou et al., 2021)
How to promote engagement with performance situations?	To recreate performance contexts that elicit realistic emotional and cognitive activation in safe, controlled conditions.	Contextualization scripts were organized hierarchically according to MPA levels (Moura and Serra, 2021), ensuring progressive emotional exposure and personalization of experience.
How and why integrate gradual exposure?	To promote progressive familiarization with MPA-inducing contexts using a validated anxiety-reduction strategy.	Grounded in Fear Emotional Processing Theory (Foa and Kozak, 1986; Foa et al., 2006) gradual exposure served as a structuring principle, supported by evidence of controlled simulations in MPA management (Williamon et al., 2014; Bissonnette et al., 2016; Aufegger et al., 2017).
How to conduct active self-regulation training?	To strengthen top-down control through alternating MPA induction and emotional regulation phases.	The training integrated controlled gradual exposure and emotion regulation approaches known to engage prefrontal cortex activity and strengthen top-down regulation of limbic responses (Ochsner and Gross, 2005; Gruzelier, 2014; Etkin et al., 2015; Paret et al., 2019; Kohl et al., 2020).
How to design anxiety-inducing and control stimuli?	To create verbal stimuli that evoke and regulate MPA in a controlled, evidence-based manner.	The stimuli were formulated based on literature on MPA and emotion regulation (Gross, 1998, 2015; Papageorgi et al., 2007; Osborne and Kenny, 2008; Clark and Williamon, 2011; Duke et al., 2011; Kenny, 2011; Glassman et al., 2016; Juncos et al., 2017; Papageorgi, 2021, 2022), integrating empirically validated strategies such as breathing and relaxation (Glassman et al., 2016; Guyon et al., 2020), attentional focus (Duke et al., 2011), biofeedback and physiological self-regulation (Barros et al., 2019; Borges et al., 2023), as well as imagery and mental rehearsal (Connolly and Williamon, 2004; Guillot and Collet, 2005; Hoffman and Hanrahan, 2012; Immonen et al., 2012; Schaefer, 2015).
How should active training evolve over time?	To guide the progressive development of emotional and physiological self-regulation.	Structured around the four-stage self-regulation model—Adaptation, Symptom Identification, Refinement, and Conditioning (Borges et al., 2023)—supported by evidence that repetition and cumulative experience consolidate self-control (Gruzelier, 2014; Paret et al., 2019)

**Figure 1:**
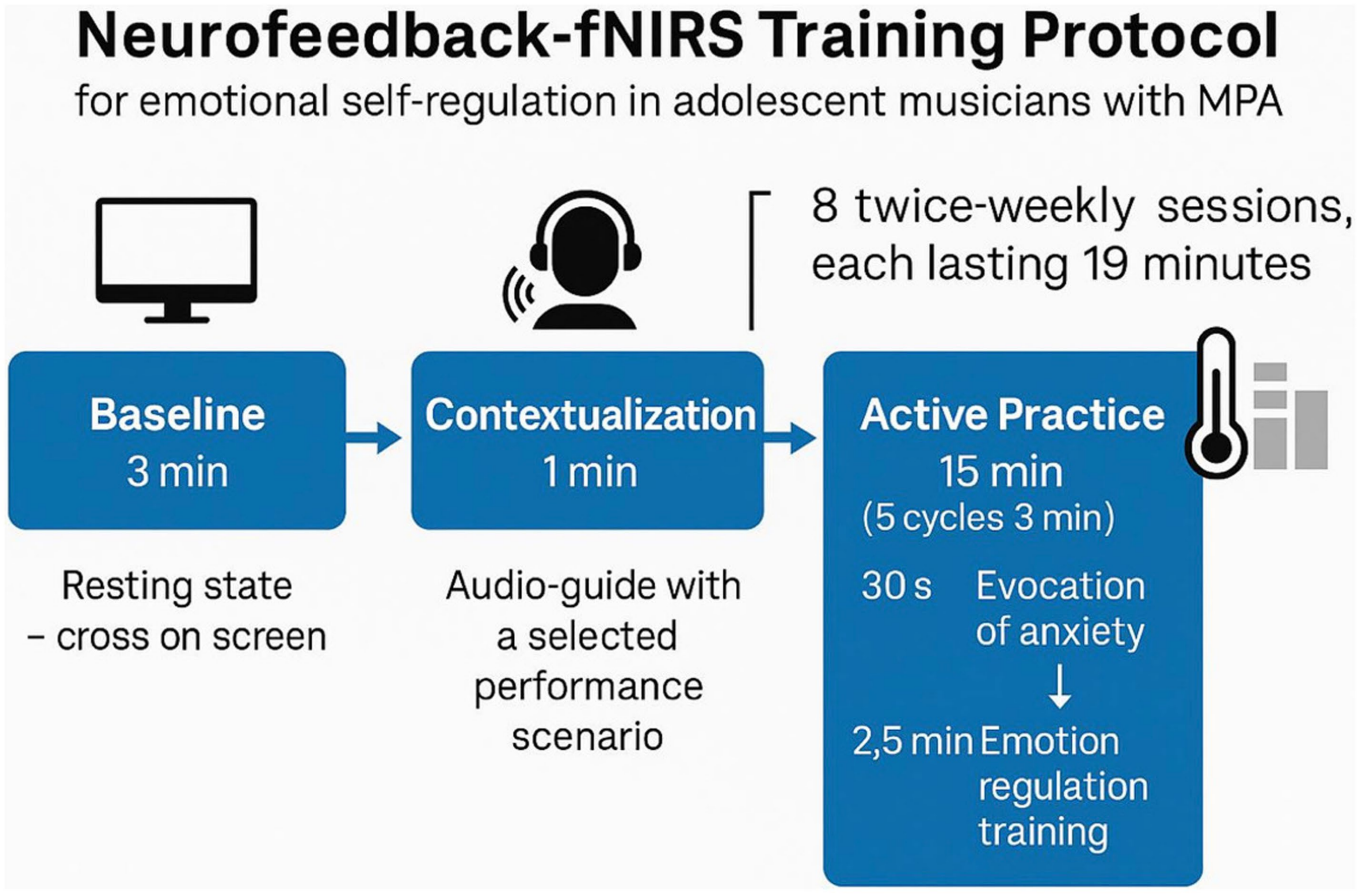
Temporal scheme of one training session, illustrating the sequence of the three phases and the cyclical alternation between evocation and regulation within the 19-min structure.

The protocol integrates three main components. First, gradual exposure was used to elicit anxiety-related cognitive and emotional states associated with musical performance, following hierarchical performance situations adapted from Moura and Serra (2021). Second, script-driven imagery was used to standardise the induction of performance-related mental states through pre-recorded auditory narratives (Shin et al., 1999; Kearns and Engelhard, 2015). Third, NFDK-fNIRS training was used to support the development of neurophysiological self-regulation through repeated cycles of evocation and control (Misaki et al., 2018; Hou et al., 2021). The full programme was structured into eight biweekly sessions designed to be brief enough (19 min each) to minimise fatigue while maintaining repeated training exposure (Kober et al., 2014; Marx et al., 2015; Kohl et al., 2020; Trambaiolli and Falk, 2023). Sessions followed a progressive four-stage sequence—Adaptation (Sessions 1–2), Symptom Identification (Sessions 3–4), Refinement (Sessions 5–6), and Conditioning (Sessions 7–8)—based on the physiological self-regulation development model proposed by Borges et al. (2023). Each session comprised three phases: baseline—resting state data collection (3 min), contextualisation—the participant listens to the musical performance scenario corresponding to their individual anxiety-inducing task hierarchy (1 min), and active training (15 min)—consisting of five consecutive three-minute blocks, alternating between 30 s of MPA induction and 2.5 min of MPA regulation (Figure 1). During active training, short verbal prompts were delivered at 30-s intervals to alternate between MPA induction and regulatory effort. This timing was defined in accordance with the haemodynamic response profile typically observed in fNIRS studies, allowing sufficient time for signal development while maintaining a structured training rhythm (James et al., 2009; Pinti et al., 2020; Yücel et al., 2021). This design ensures a balanced alternation between induction and MPA control, supporting gradual learning of neurophysiological self-regulation (Blume et al., 2017; Hou et al., 2021). Stimuli were presented following a controlled randomization scheme to ensure variation across sessions and reduce habituation effects.

### 2.3 Protocol operationalisation

This stage focused on transforming the conceptual structure of the NFDK-fNIRS training into a standardised and executable protocol, including specifying the materials, programming the experimental sequence, integrating the acquisition and feedback systems, and defining implementation procedures to ensure consistency across participants and sessions.

#### 2.3.1 Materials

##### 2.3.1.1 Software

PsychoPy (v2024.2.4), was used for stimulus presentation, event randomisation, and temporal control with the fNIRS system. TurboSatori (Brain Innovation, v2020.2.2) generated real-time visual NFDK in thermometer format. Aurora fNIRS (NIRx, v2023.9.6) was responsible for acquiring and visualising hemodynamic data from the NIRSport2 system. Together, these platforms enabled synchronised stimulus delivery, physiological acquisition, and feedback presentation.

##### 2.3.1.2 Hardware

Data were acquired using a NIRx NIRSport 2 System with an 8 × 8 frontal optode montage positioned over the prefrontal cortex. Source–detector pairs spaced approximately 30–35 mm apart, targeting the right dorsolateral prefrontal cortex (rdlPFC). Signals were sampled at 10.2 Hz. Auditory stimuli were delivered using Beyerdynamic DT 770 Pro headphones (80 *Ω*). A computer with sufficient processing capacity to run PsychoPy, Aurora fNIRS, and TurboSatori simultaneously supported the experimental setup.

##### 2.3.1.3 Instrument

The adapted “Ten-Item List” (Last, 2006) for MPA (Moura and Serra, 2021) expanded to 19 performance situations was used to rank anxiety-provoking contexts and guide individualised exposure during training.

##### 2.3.1.4 Audio guides

Two sets of standardised audio guides were developed: *(a) contextualisation audio guides*, based on the 19 performance situations described by Moura and Serra (2021), corresponding to contexts that elicit MPA (scripts available in Supplementary material 1); *(b) active training audio guides* – Developed from the theoretical framework and consisting of a set of 240 short verbal phrases, including 40 anxiety-evoking and 200 regulation (control) statements (scripts available Supplementary material 2).

#### 2.3.2 Operational procedures

Three sets of procedures were defined to ensure standardisation and implementation quality: stimulus development, system integration, and quality control.

##### 2.3.2.1 Stimulus development

verbal scripts were developed from the reference literature and refined with the support of artificial intelligence tools to ensure linguistic consistency and thematic coherence. Audio guides were professionally recorded in MP3 format (44.1 kHz, 16-bit, mono), with precisely pre-programmed durations (e.g., 30 s or 1 min), ensuring synchronisation with experimental events. The selected voice was judged by the research team to be neutral, clear, and rhythmically stable.

##### 2.3.2.2 System integration

PsychoPy controlled the presentation of visual instructions, contextualisation scripts, and induction/regulation prompts, while also time-stamping experimental events and storing task data (PsychoPy script available through GitHub at: https://github.com/pedrorribeiro/MUSA-Neurofeedback-training). Real-time NFDK-fNIRS was displayed as a dynamic thermometer via TurboSatori, using hemodynamic signals acquired through Aurora fNIRS. This configuration enabled continuous interaction between task execution, physiological monitoring, and feedback delivery.

##### 2.3.2.3 Quality control and implementation conditions

An operational manual was developed to standardise equipment setup, software configuration, participant instructions, and signal verification. Additional precautions were adopted to reduce common sources of noise and disruption, including control of ambient light, use of closed-back headphones, and door signage to minimise interruptions during data collection.

### 2.4 Participants and setting

The study included three female adolescents with previous experience in public performance contexts. Participants were selected based on high levels of MPA, identified through an initial screening process. The inclusion criterion consisted of obtaining a total MPAI-A score above the mean observed for female adolescents (*M* = 55.84), used as a reference value derived from a screening study including 513 music students (62% female) from 25 specialized music education institutions in Portugal. All three participants met this criterion, presenting scores of 56 (Participant 1, age 12), 58 (Participant 2, age 13), and 73 (Participant 3, age 12).

Participant 1 had been studying a keyboard instrument for 36 months at the time of the study. In the most recent evaluation period, she received the highest possible grade (5 out of 5), and reported a weekly practice routine of four hours. Over the previous school year, she had taken part in six performances. Participant 2, a string instrument player with 48 months of formal study, received a grade of four in her last evaluation. She dedicated six hours per week to individual practice and accumulated five performance experiences throughout the preceding school year. With the longest instrumental trajectory among the three, Participant 3 had studied a keyboard instrument for 84 months. Her most recent evaluation also yielded a grade of four, and she reported practicing five hours weekly. During the last school year, she participated in seven performances.

Given the exploratory nature of this proof-of-concept study, a small and homogeneous sample was intentionally used to prioritize technical feasibility testing and protocol refinement before larger-scale evaluation (Skivington et al., 2021). The study was approved by the local ethics committee (ref. CETCH2023-33), and written informed consent was obtained from participants and their legal guardians. Sessions were conducted during regular school hours in music schools, thereby preserving ecological relevance to the participants’ usual learning and performance environments.

### 2.5 Training procedures

The proof-of-concept implementation focused on three domains aligned with the study aims: (a) technical feasibility concerned signal stability, synchronization, and reliability of data acquisition reability; (b) procedural applicability concerned calibration consistency, session duration, and participants’ understanding of the instructions; and (c), functional coherence concerned whether the structure of the training appeared compatible with the intended process of self-regulation learning.

At the beginning of the first session, participants completed the Ten-Item List adapted for MPA (Moura and Serra, 2021), which allowed for the organization of gradual exposure according to each individual’s anxiety profile. For proof-of-concept purposes, the implementation comprised four sessions representing a compact version of the full eight-session protocol (Borges et al., 2023), while preserving the same internal structure of baseline, contextualisation, and active training.

At the beginning of each session, the instructors installed the fNIRS system, checked participant comfort, and completed signal calibration. Neutral conversation was used during setup to facilitate adaptation to the equipment. Participants then received instructions corresponding to the relevant training stage. Sessions were scheduled twice weekly, with at least one day between them.

Because the purpose of this phase was feasibility testing rather than efficacy evaluation, repeated subjective MPA measures were not administered during the sessions. At the end of each session, participants were invited to share brief reflections on their training experience, including perceptions of difficulty, comfort, and engagement. These observations were recorded by the instructors and integrated into the *reflective research journal*, providing qualitative insight to inform subsequent refinement of the protocol.

### 2.6 Proof-of-concept evaluation

In line with the proof-of-concept framework, the protocol was evaluated used two complementary sources of evidence: (a) a *reflective research journal* (Ortlipp, 2015), which documented implementation-related observations and decisions, and the (b) neurophysiological data obtained through NFDK-fNIRS, which provided objective indicators of hemodynamic modulation during training.

#### 2.6.1 Reflective research journal

During implementation, the instructors maintained a reflective research journal (Ortlipp, 2015) to document observations related to technical functioning, procedural flow, participant adaptation, and contextual conditions. Entries were written after each training session and included notes on equipment setup, signal quality, participant engagement, perceived effectiveness of stimuli, and contextual factors affecting implementation. The notes were analysed using a reflexive thematic approach (Ortlipp, 2015; Braun and Clarke, 2019), allowing the identification of relevant patterns for the evaluation of the proof of concept and guiding conceptual and operational refinement of the protocol.

#### 2.6.2 NFDK-fNIRS

**Figure 2:**
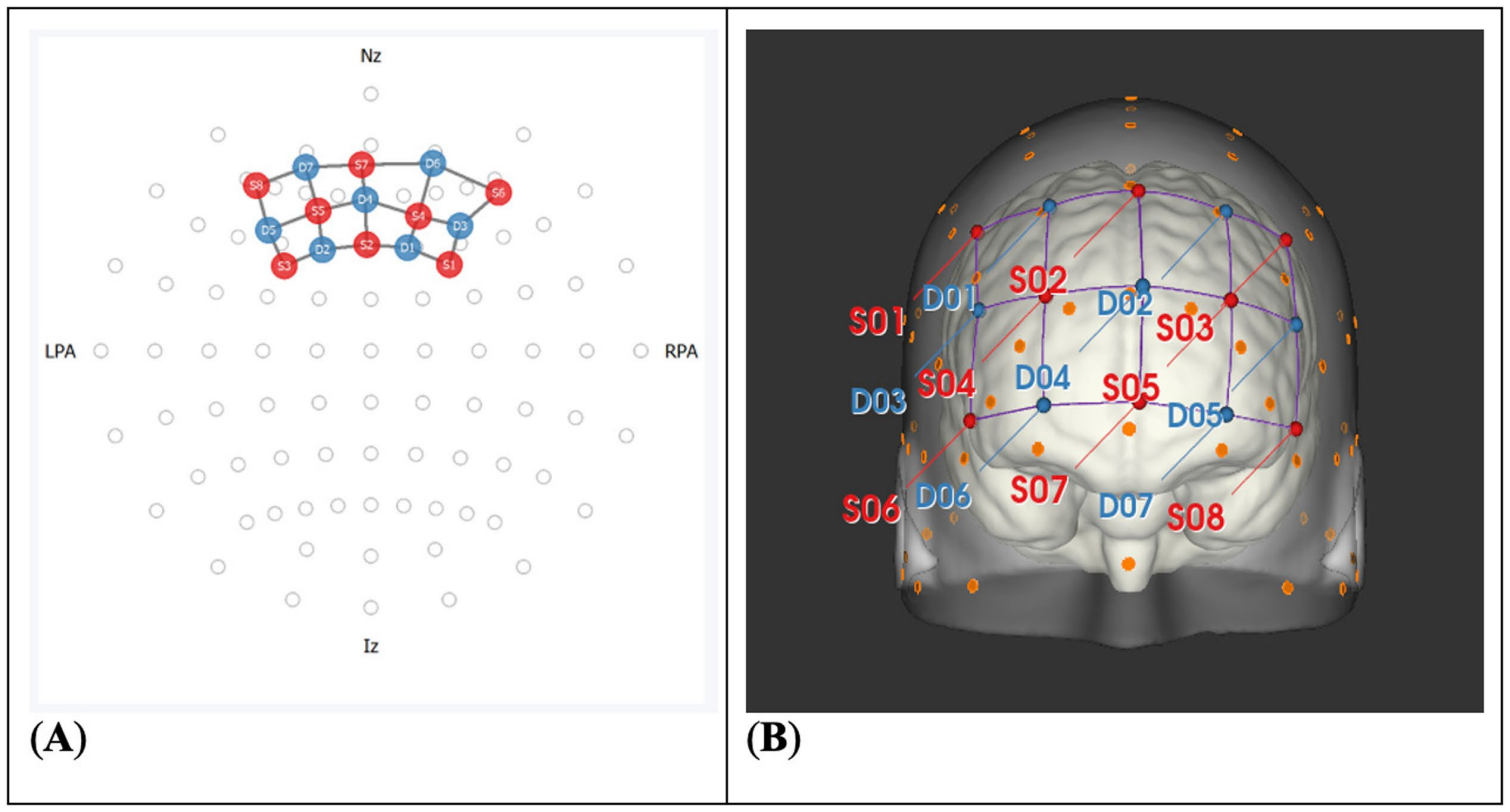
Standard headband 8×8 montage used for neurofeedback and data collection. **(A)** 2D representation of the montage. **(B)** Scalp and cortical projection of the montage.

NFDK-fNIRS was conducted using the NIRx NIRSport 2, with a standard headband 8×8 montage, which can be seen in Figure 2. Data was collected at 10.2 Hz sampling rate. Brain Innovation software TurboSatori was used to display the NFDK signal as a thermometer. Turbo-Satori (v2020.2.2) was set to filter the data with a bandpass filter with a stopband of 0.01 to 0.3 Hz. Data from all channels was simultaneously collected via Aurora fNIRS from NIRx (v2023.9.6). PsychoPy (v2024.2.4) was used for stimulus presentation and data synchronization. At the start of the protocol, a resting state baseline was collected for every participant, where they were asked to sit still while looking at a white fixation cross on a black background.

The fNIRS data collected was thoroughly pre-processed. Initially signals were cropped to exclude any signal epochs which happened before the start of the Baseline and after the end of the NFDK training. A finite impulse response, zero-phase, bandpass filter of order 1000, leveraging a Blackman window setup was used to remove slow drifts and high frequency noise, while keeping the physiological fNIRS range (0.01 to 0.1 Hz). Wavelet denoising was conducted by decomposing signals with a Symlet 4 wavelet type. The signal was decomposed in 10 levels, then detail coefficients at levels D1 through D5 were removed to ensure the removal of movement and other high frequency artifacts, preserving slower hemodynamic trends. The signals were then converted to optical density, and subsequently to haemoglobin concentration through the modified Beer–Lambert law, with a differential pathlength factor of 7.25 at 760 nm, and 6.38 at 850 nm, and extinction coefficients of 645.5 and 1669.0 for oxy-haemoglobin calculation at 760 and 850 nm, respectively. Finally, the signals were mean-centered (demeaned) and normalized based on their root-mean-square amplitude.

After preprocessing, the signals were epoched for the stressor and control events, with 15 s epochs staring at event onsets. The epoch to mean energy ratio was calculated for each epoch and then epochs of the same event were averaged. Energy was defined as per Equation 1:


$$E=\sum_{n=-\infty }^{\infty }{|x(n)|}^{2}$$ (1)

## 3 Results

Based on observations documented in the reflective research journal (Ortlipp, 2015), the analysis identified five main dimensions reflecting the feasibility, coherence, and applicability of the NFDK-fNIRS training in a school environment (Braun and Clarke, 2019).

### 3.1 Technical feasibility and operational stability

The system demonstrated overall stability and technical coherence throughout the sessions, following minor signal corrections and calibration adjustments, which included verification of signal traces, repositioning of optodes, and adjustment of the cap to optimize signal acquisition. It was observed that synchronization between software and hardware components must follow a specific logical activation sequence to ensure proper system functioning; any deviation from this order or absence of a standardized procedure can lead to operational failures and bugs during training. The advance preparation of equipment and the use of headphones optimized setup time and contributed to session stability. The maximum time for each session was 45 min, including installation, instruction, calibration, and system configuration, ensuring full functionality before the start of each session. An execution guide was developed and applied to standardize the procedure and ensure the success of the training.

### 3.2 Participant adaptation and self-regulatory engagement

Participants showed progressive familiarization with the protocol, reflected in greater adherence, cooperation, and accuracy in following the instructions. The use of headphones supported concentration and the comprehension of auditory stimuli. Progressive improvement was observed in recognizing anxiety-inducing stimuli and applying MPA control strategies, accompanied by consistent variations in the feedback thermometer across sessions.

### 3.3 Perception of stimuli and suitability of training content

Participants reported that the induction audios were effective in facilitating and guiding the mental simulation of performance situations. However, one participant reported discomfort in response to specific control stimuli, as these were associated with strategies she had previously attempted without success. Rather than representing a failure of the protocol, this reaction highlights the importance of individual differences in the perceived relevance and emotional resonance of regulation cues. Such differences are well documented in the literature on emotion regulation and NFDK learning. Methodologically, this observation underscores the need for flexible personalization of control stimuli, particularly in early training phases, to prevent the reactivation of maladaptive associations and to optimize engagement and learning.

### 3.4 Transfer to performance contexts

During the implementation period, two participants took part in public performances within their school settings and spontaneously self-reported having applied the control strategies practiced during training. Observations recorded during these events indicated the presence of self-regulatory behaviours consistent with those developed in the sessions.

### 3.5 Contextual conditions

Several contextual constraints inherent to the school environment were recorded, including external noise from neighbouring rooms, occasional interruptions, and limitations of time and space. Considering the versatile nature of the equipment and the protocol, it is essential to anticipate their implementation in environments without full control over these variables. Measures such as the use of noise-blocking headphones, door signage during sessions, and prior communication with school staff are fundamental to minimizing interruptions. These observations were progressively incorporated throughout the implementation process, guiding adjustments aimed at optimizing experimental conditions and preserving the ecological validity of the study. Qualitative observations provide relevant and coherent evidence to support the consolidation and refinement of the training protocol in real educational contexts.

The qualitative observations were complemented by neurophysiological analyses, allowing for a more comprehensive understanding of the self-regulation process. Whereas the reflective journal data illuminated the experiential and behavioural dimensions of adaptation and engagement, the fNIRS results provided an objective counterpart, revealing cortical modulation patterns consistent with the progressive development of top-down regulatory mechanisms. This integration of subjective and physiological levels of analysis reinforces the multidimensional coherence of the protocol and its potential to capture both the experiential and neural correlates of MPA regulation.

**Figure 3:**
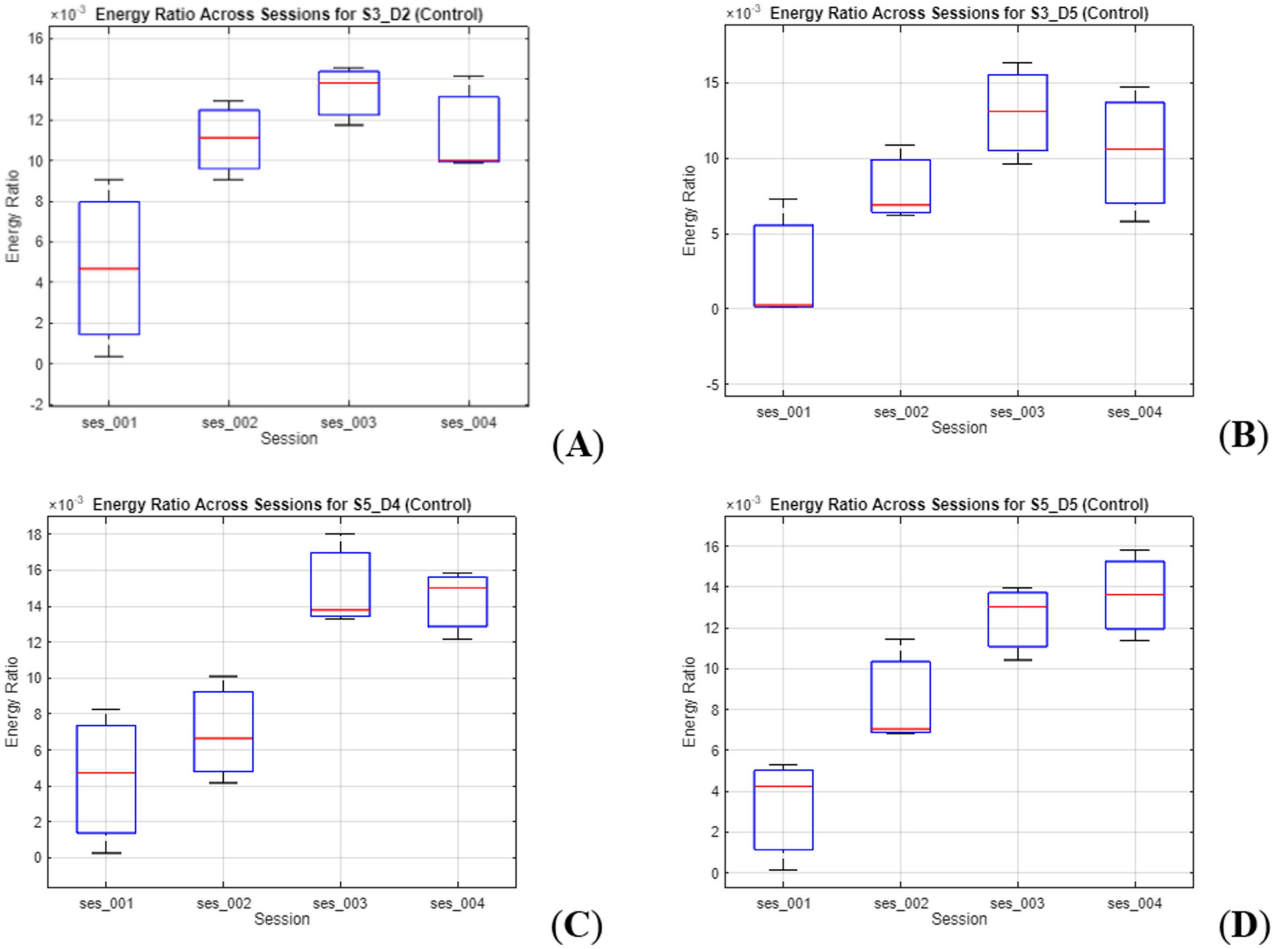
Energy ratio across sessions for selected prefrontal channels during the control condition. **(A)** Channel S3–D2, **(B)** Channel S3–D5, and **(D)** Channel S5–D5 correspond to regions within the left dorsolateral prefrontal cortex (dlPFC), while **(C)** Channel S5–D4 lies within the dorsomedial prefrontal cortex (dmPFC). All four channels display session-dependent increases in energy, with the strongest and most consistent growth observed in S5–D5. Channel S5–D4 also shows a substantial increase between sessions 2 and 3, despite a plateau in later sessions. These findings suggest progressive engagement of lateral and medial prefrontal areas throughout the intervention.

Of the prefrontal channels assessed through fNIRS, only 4 had observable differences in activation patterns in energy ratio, across the four sessions, all of which presented changes in the control condition. Channels S3-D2, S3-D5, and S5-D5, encompassing the left dorsolateral prefrontal cortex (left dlPFC), and channel S5-D4 partially encompassing the dorsomedial prefrontal cortex (dmPFC). Typically, energy in these areas tended to increase up to session 3, before plateauing in session 4. The exception was channel S5-D5, which kept rising up to session 4. This can be seen in Figure 3, as shown by boxplots of the energy ratio per session.

## 4 Discussion

This proof-of-concept study examined whether a NFDK-fNIRS protocol for MPA could be conceptually developed, operationalised, and implemented in educational settings for adolescent musicians. To provide a coherent interpretative structure, the findings are discussed across three interrelated dimensions: (1) conceptual coherence of the protocol, (2) feasibility in educational settings, and (3) functional coherence as a self-regulation training framework. Overall, this structure aims to provide preliminary support for the internal consistency of the protocol across these dimensions, while also revealing variability in participants’ responses that warrants further refinement. This perspective allows the discussion to move from theoretical assumptions to their practical translation and observed effects, maintaining continuity across the study’s design.

Within this framework, the conceptual grounding of the protocol represents the first level of analysis. The findings suggest that the protocol is theoretically coherent with current models of emotional self-regulation, particularly in its integration of gradual exposure and top-down control mechanisms. The protocol was conceptually grounded in the regulation of MPA as a self-regulatory learning process, targeting the prefrontal cortex (dlPFC/dmPFC) as a plausible top-down training site and privileging ecological validity. Rather than treating MPA solely as a disruptive condition, the protocol frames it as a dynamic and trainable process, which constitutes an important conceptual shift in the field.

The integration of gradual exposure and self-regulation training, guided by standardized audio scripts, was intended to provide a structured yet flexible framework for engagement. This structure aimed to promote consistency across participants while still allowing individual engagement with the task. Importantly, this balance between standardization and flexibility reflects an attempt to align experimental control with ecological validity, which is a central challenge in applied neurofeedback research. Within this framework, the use of fNIRS extends this conceptual rationale by enabling the monitoring of cortical activity in ecologically valid settings, given its portability, cost-effectiveness, and tolerance to movement (Tuscan et al., 2013; Fishburn et al., 2014). Accordingly, the protocol frames MPA as a potentially trainable phenomenon, promoting cognitive and physiological strategies that may be transferable to real performance situations (Ochsner and Gross, 2005; Linden et al., 2012; Gruzelier, 2014; Gross, 2015).

Building on this conceptual foundation, the next level of analysis concerns the feasibility of translating the protocol into real-world educational settings. The operationalisation phase translated these conceptual decisions into technical and procedural components. Alternating between evocation and regulation phases appeared to be suitable for the hemodynamic kinetics of fNIRS and to support deliberate self-regulatory practice (James et al., 2009; Pinti et al., 2020; Yücel et al., 2021). The use of audio guides aimed to ensure temporal and semantic standardization, reducing inter-subject variability in line with script-driven imagery and emotion-oriented NFDK paradigms (Shin et al., 1999; Kearns and Engelhard, 2015; Zotev et al., 2018; Hou et al., 2021). The results suggested technical feasibility, signal stability, and progressive procedural fluency, indicating the suitability of the protocol to school environments and its potential to support self-regulatory autonomy during adolescence (Ros et al., 2014; Paret et al., 2019). These findings suggest that the proposed structure is not only implementable, but also compatible with the practical constraints of educational contexts. Preliminary patterns of prefrontal activation may indicate engagement of regions implicated in emotional regulation, aligning with prior evidence. However, these observations remain exploratory and should be interpreted with caution given the small sample size and absence of formal statistical validation. While fNIRS appeared to offer a suitable balance between ecological validity and neural specificity, its sensitivity to superficial hemodynamic artifacts and limited spatial resolution should be acknowledged. Future iterations may integrate short-separation channels and physiological baselines to strengthen neural specificity.

The final level of analysis concerns whether the protocol operates in a manner consistent with its intended function, namely, the training of self-regulation. Participants showed growing adherence, understanding of instructions, and intentional application of control strategies, which may reflect progressive engagement with the self-regulation process over time. Physiological responses displayed modulation across sessions, while contextual factors such as noise, interruptions, and time constraints appeared not to substantially affect implementation, indicating that the protocol can be implemented under real-world educational conditions. The convergence between behavioural adaptation, subjective reports, and neurophysiological modulation provides preliminary support for the functional coherence of the protocol. This alignment suggests that the training structure may successfully engage the mechanisms it was designed to target, although these interpretations remain exploratory.

Focusing on the neurophysiological dimension of the findings, results obtained through fNIRS revealed observable energy-ratio changes in four frontal channels—three in the left dlPFC (S3–D2, S3–D5, S5–D5) and one in the dmPFC (S5–D4). Although *post hoc* comparisons were not conducted due to the exploratory nature and small sample size, session-wise differences may suggest longitudinal modulation of prefrontal activation across training. The progressive increase in activation appears to be broadly aligned with previous literature linking the dlPFC and dmPFC to cognitive reappraisal, emotional monitoring, and attentional control (Ball et al., 2013; Buhle et al., 2014; Kohl et al., 2020; Zhao et al., 2024). From an interpretative perspective, this pattern may reflect top-down cortical learning and gradual recruitment of higher-order prefrontal circuits, as often reported in emotion-related NFDK protocols (Li et al., 2025; Zeng et al., 2025; Zotev et al., 2018). These observations can be interpreted as indicating that the protocol may engage neural mechanisms associated with self-regulatory processes, although such interpretations remain preliminary.

Expanding on these neurophysiological observations, the increasing engagement of the left dlPFC, a region implicated in emotional regulation and cognitive reappraisal, could indicate neuroplastic adaptation and emerging self-regulatory learning. The minor reduction in prefrontal activation observed in the final session may be associated with cognitive fatigue resulting from repeated training exposure or the gradual automatization of self-regulation strategies, as sometimes observed in short NFDK training protocols (Paret et al., 2019). Within this context, NFDK paradigms are widely conceptualized as learning-based processes in which individuals gradually acquire voluntary control over brain activity through repeated feedback exposure (Sulzer et al., 2013; Sitaram et al., 2017). Changes in neural activation across sessions are often interpreted as markers of regulatory learning dynamics. In line with this perspective, increased recruitment of the left dlPFC has been associated with the mitigation of internalizing disorders, including performance anxiety (Wu et al., 2024), and with the alleviation of emotional pain (Alizadehgoradel et al., 2024). This supports the functional relevance of this region in emotion regulation. Previous findings also indicate that NFDK training can improve emotion-regulation skills after a few sessions, suggesting the trainability of the dlPFC (Yu et al., 2021). Similarly, the dmPFC contributes to cognitive and self-reflective reappraisal strategies, in cooperation with the dlPFC, and its hypoactivation has been associated with generalized anxiety disorder (Ball et al., 2013; Buhle et al., 2014). Importantly, the convergence between participants’ reports of increased emotional control and the observed modulation of prefrontal activity points to a potential coherence between experiential and physiological dimensions of self-regulatory learning. While the reflective journal captured participants’ growing familiarity with anxiety-inducing stimuli and their intentional application of control strategies, the fNIRS data revealed progressive engagement of prefrontal regions implicated in cognitive regulation. This alignment between subjective and neurophysiological indicators may be interpreted as an early sign of functional integration within the training process. However, the close researcher–participant interaction, while beneficial for engagement, may have introduced expectancy effects. In addition, individual differences in motivation, baseline anxiety, and musical experience likely contributed to within-subject variability.

Across all phases, the study offers preliminary signs of coherence between theory, method, and evidence. Subjective (reduced apprehension and greater self-control), behavioral (fluency and autonomy), and physiological (prefrontal modulation) indicators collectively point to the adequacy of the protocol for the target population and its potential as a training tool for emotional self-regulation in young musicians (Blume et al., 2017; Misaki et al., 2018; Hou et al., 2021). From a broader perspective, this convergence across multiple domains may reflect an initial alignment between the conceptual rationale of the protocol and its practical implementation. Despite the exploratory design and limited sample size, the results support its feasibility and applicability in school contexts. Future studies should include controlled trials, longitudinal follow-ups, and the integration of multivariate measures (fNIRS, physiological markers, and MPA scales) to strengthen convergent validity and explore its sustainable integration into psychoeducational programs aimed at well-being and musical performance (Moore et al., 2015; Skivington et al., 2021; Schmidt et al., 2024).

Some limitations must be considered when interpreting these findings. First, the proof-of-concept implementation involved a very small sample, which limits the generalizability of the observations and precludes statistical inference. Second, subjective measures of MPA were not collected during the training sessions, preventing a more precise comparison between perceived emotional change and neurophysiological modulation. Third, although fNIRS provides ecologically suitable measurement conditions, it has limited spatial resolution and is sensitive to superficial physiological artefacts. These factors reinforce that the present findings should be interpreted as preliminary indicators of feasibility and conceptual coherence rather than evidence of intervention efficacy.

Future studies may explore extended training protocols of up to eight sessions, as proposed in the physiological self-regulation development model (Borges et al., 2023), as well as alternative formats involving shorter but more frequent sessions. It is also expected that the protocol will be replicated with larger and more diverse samples, including male adolescent musicians to address gender homogeneity, as well as participants from different age groups and musical contexts. A 1-month follow-up may also be conducted to assess the retention of self-regulation skills. Future work may incorporate validated self-report measures of MPA and related constructs such as flow, alongside peripheral physiological indicators of anxiety.

Overall, these exploratory findings suggest that the proposed NFDK-fNIRS protocol is technically feasible and conceptually coherent as a preliminary training framework for self-regulation of MPA. However, further validation with larger samples and controlled study designs is required before conclusions regarding efficacy can be drawn. More importantly, they provide an initial integrative framework linking theory, method and application in the study of MPA self-regulation, which may inform future controlled investigations.

## Data Availability

The raw data supporting the conclusions of this article will be made available by the authors, for research purposes, upon request.
