# Supplementary material for: From concept to proof: developing a neurofeedback-fNIRS protocol to train self-regulation for music performance anxiety in adolescent musicians
Source: Front Psychol. 2026 Apr 29;17:1746761. doi: 10.3389/fpsyg.2026.1746761 (PMC13170606; doi:10.3389/fpsyg.2026.1746761)
Supplement: Supplementary file 1 [file Supplementary_file_1.DOCX]

Supplementary Material 1

# Text that originated the audio guides

Below are the induction texts for the 19 performance situations proposed by Moura (2019):

- **Playing or singing a piece for your parents**

Imagine yourself in a warm, familiar room. Your parents are sitting in comfortable chairs, watching you attentively. Soft light enters through the window, bathing the space in golden tones. You adjust the position of your instrument and feel its weight in your hands. The ambient noise is low, perhaps just the ticking of a distant clock or the soft sounds of a quiet home. The familiar smell of the room brings a sense of comfort. You correct your posture and take a deep breath. You are ready, with the sheet music in front of you, prepared to guide each note. The space falls silent as you prepare to begin the performance, every detail of the room clear in your mind.

- **Playing or singing solo in the final recital**

Visualise yourself backstage in an auditorium. You hear the soft murmur of the audience and the muffled sounds of the other performers. A heavy curtain separates you from the stage. When you hear your name called, you pull the curtain aside and walk to the centre of the stage. The lights focus on you, illuminating the space around you while the audience remains in the shadows. You feel the firm ground beneath your feet and the texture of the wooden floor. The sheet music is correctly positioned on the stand. Looking out into the auditorium, rows of seats extend in front of you. The room is silent, and you adjust yourself into the correct position to start your performance.

- **Concert with the orchestra**

Imagine yourself seated in the middle of an orchestra, surrounded by other musicians with their instruments ready. You see the bows of the violins aligning, wind players adjusting their reeds, and percussionists preparing the final details. The stage lights reflect off the instruments, creating subtle gleams. The conductor steps onto the podium and lifts the baton, signalling the start. You look at the sheet music in front of you, each note clear and precise. The pages are well secured, and you are in the ideal position to begin. The sound of the first chords blends together as the room fills with harmony. You follow the perfect synchrony among the musicians, attentive to the conductor’s cues.

- **Recording a piece in the classroom**

You are sitting in your classroom, with the recorder positioned in front of you. The room is silent, the only sound being the low hum of electrical equipment. The white light from the lamps softly illuminates the walls and floor. You are ready, with the sheet music open at the exact point where the piece begins. The recorder shows a red light, indicating that it is on. Your teacher sits nearby, watching attentively. You adjust your posture, ensuring everything is in the right place. The windows are closed, blocking any external noise. The space is prepared to capture every sound with precision.

- **Instrument evaluation exam**

Visualise yourself in a quiet, organised room. A teacher sits in front of you at a table with a clipboard and several sheets of paper. You are in the ideal position. The soft light in the room illuminates the sheet music before you. Every line and symbol is clearly visible. The teacher gives you a slight nod to begin. The environment is formal, with light-coloured walls and acoustics that allow every sound to echo gently. You adjust your position and prepare to start the piece. Every detail of the room is clear in your mind—from distant footsteps in the hallway to the texture of the chair beneath you.

- **Playing in the large auditorium for the teacher (without an audience)**

You walk to the centre of a large, silent auditorium. The rows of chairs are empty, and the space echoes with your footsteps. Only your teacher sits in the front rows, watching attentively. The soft stage lighting shines on you and your instrument. You adjust the music stand and check your positioning. The auditorium has clear acoustics, allowing any sound to spread easily. The silence is deep, broken only by the sound of the wooden stage beneath your feet. You take a deep breath and focus your attention on the piece you will perform.

- **Playing for a classmate during a lesson with the teacher**

Imagine yourself in a classroom. Your classmate sits a few metres away, with the teacher observing beside them. The environment is calm, with only the soft rustling of sheets and the sound of breathing filling the space. You hold your instrument firmly and adjust the position of the sheet music. The room is softly lit, and you notice details of the surroundings—the boards on the wall, the organised desks, the gentle shine of the instruments. Your classmate watches attentively, waiting to hear your performance. You position your fingers correctly and prepare to begin.

- **Playing for a different teacher in class**

Imagine yourself in a classroom different from your usual one. In front of you sits a teacher you do not know well, holding the sheet music. The room feels slightly unfamiliar—the windows may face a different direction, or the furniture might be arranged uniquely. You are ready and adjust your posture. The teacher signals for you to begin. Natural light enters softly through the window, illuminating the sheet music in front of you. Every detail of the room is clear—the distant sound of footsteps in the hallway, the faint smell of polished wood. You position yourself and prepare to start, fully focused on the performance.

- **Playing a duet with the teacher during a recital**

Visualise yourself on a stage prepared for a recital. You stand beside your teacher, both ready to play a duet. The chairs are placed closely, and your sheet music is open side by side. The stage lights cast a gentle glow over the space. You hear the silence of the auditorium and the soft sound of both your breaths as you prepare to begin. Your teacher makes brief eye contact and gives a small nod. Your instruments are tuned and ready. The atmosphere is focused, and the stage feels like a space reserved solely for the music you are about to play.

- **Playing for an unfamiliar audience**

Imagine yourself stepping onto a stage in an auditorium filled with people you have never seen before. The lights focus on you while the audience remains in darkness. You perceive the silhouettes of the listeners and the low murmur that gradually fades. The space has clear acoustics, and every sound seems amplified. Your sheet music is well positioned on the stand, and your instrument feels steady in your hands. You adjust your posture and check your finger position. The atmosphere is one of quiet expectation. Everything is ready for you to begin, and the room awaits, fully attentive to the music you are about to play.

- **Playing or singing in a place outside school**

Imagine yourself in a new place, different from your usual classroom. It could be a small community room, a library, or an event hall. The walls are decorated differently, and there are new smells and subtle sounds around you. Some people are seated, waiting for your performance. Light enters the room differently through the windows, casting unique shadows on the floor. You sit or stand in your position, focus yourself, and look at the sheet music on the stand. The environment is unfamiliar, but everything is ready for the performance. You take a deep breath, attentive to every detail of this new space.

- **Recording a piece in the large auditorium**

Imagine yourself in a large, empty auditorium prepared for a recording session. Microphones are positioned around you, and a technician adjusts the final details of the equipment. Empty rows of chairs extend before you, and the stage lighting creates an atmosphere of focus. Your footsteps echo through the silent space. You sit or stand in place, adjust the sheet music, and concentrate. The red recording light is on, indicating everything is ready. The environment is perfectly prepared to capture every note with precision. You take a deep breath and prepare to begin.

- **Playing a duet with a classmate during a recital**

Imagine yourself on stage next to a classmate. You are both ready to begin your performance in a recital. The chairs are positioned close together, and your sheet music is open on the stands. The stage lighting illuminates both of you as the audience watches silently. You settle yourself, focus, and look at your colleague, waiting for the signal to begin together. You both breathe in sync, ready for each note to intertwine precisely. The ambient sounds fade into total silence as you concentrate on the piece that is about to begin.

- **Playing in a quartet during a recital**

Imagine yourself seated on stage with three classmates, ready to perform a quartet. Everything is organised and prepared. The sheet music is arranged on stands, forming a small circle between you. The environment is filled with the attentive silence of the audience. The conductor or group leader gives a small gesture to signal the start. You adjust your breathing and prepare to enter in perfect harmony. Each sound blends with your colleagues’, creating a rich, unified texture. Feel the connection between all the musicians as the music unfolds.

- **Playing or singing for classmates in the FM/Musical Initiation class**

Imagine yourself in a classroom with your Musical Training classmates. They sit in chairs waiting to hear you. The room is lit by natural light entering through the windows. The teacher is beside you, observing attentively. You adjust your posture and move to your space. The sheet music is open in front of you, each line and note clear. Your classmates watch silently, following your every movement. The room is ready for you to begin, with all details surrounding you.

- **Playing or singing in a masterclass at school**

Imagine yourself in a masterclass room at your school. The guest professor sits in front of you, and your classmates are gathered around, ready to observe. The room is spacious and well lit, with sound echoing slightly off the walls. You stand in the centre with the sheet music open at the correct point. The professor gestures for you to begin. The atmosphere is one of total attention, with all eyes turned toward you, ready to hear the first sounds.

- **Playing or singing in a masterclass outside of school**

Imagine yourself in a masterclass room outside your school. It is a new space, with details you are not familiar with, perhaps, heavy curtains, tall windows, or differently decorated walls. The guest professor stands before you, and other students sit around watching attentively. You adjust your posture and position yourself. Soft light enters through the windows, creating an atmosphere of focus. The professor gives a sign to begin, and the room falls silent, prepared to listen to your performance.

- **Playing or singing for a teacher and their students in their classroom**

Imagine yourself in a classroom you know well. The chairs and music stands are arranged in a familiar way, and natural light enters gently through the windows. The teacher sits at the front with an attentive expression, and around them are other students watching. They sit in a semicircle, all close by. You stand or sit in the centre, fully prepared. The ambient noise is low—perhaps the soft rustling of papers or a distant sound from the corridor. The sheet music is open at the exact starting point. You adjust your posture and check the music stand. The space, though familiar, is focused entirely on you and the music you are about to begin. The attentive eyes of your classmates and the teacher follow your every movement.

- **Playing or singing in the Ensemble/Orchestra class for classmates**

Imagine yourself in the Ensemble or Orchestra class. The room is large, with rows of chairs and instruments resting on their stands. Your classmates are around you, each adjusting positions, warming up their voices, or tuning strings. The teacher stands at the front, holding the baton or making final adjustments to the score. There is a soft murmur of conversation and the sounds of instruments being prepared. You are in your place, with the sheet music open at the correct point. The room falls silent when the teacher signals to begin. The walls have music posters and shelves with instrument cases. The sound of restrained breathing and the subtle shine of the lights reflect in the space. You stay focused and attentive to your entry, ready to perform in harmony with your classmates around you.

**
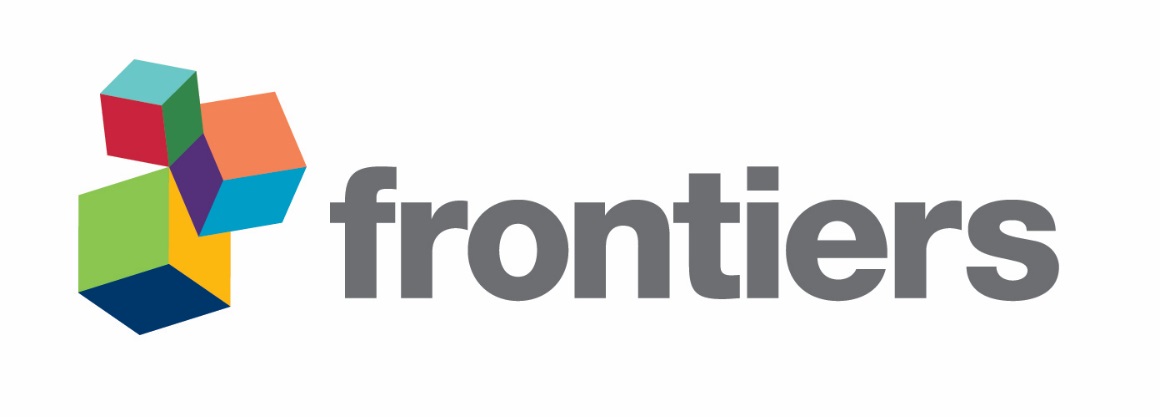
**
