# Supplementary material for: From concept to proof: developing a neurofeedback-fNIRS protocol to train self-regulation for music performance anxiety in adolescent musicians
Source: Front Psychol. 2026 Apr 29;17:1746761. doi: 10.3389/fpsyg.2026.1746761 (PMC13170606; doi:10.3389/fpsyg.2026.1746761)
Supplement: Supplementary file 2 [file Supplementary_file_2.DOCX]

Supplementary Material 2

# Anxiety and Control Inducers

The tables below present a detailed and organised structure for each session, making it easier to understand how the training sessions are structured.

## Session 1: Adaptation – Identifying Self-Regulation Processes

| **Time** | **Stress induction phrase** | **Time** | **Control induction phase** |
| --- | --- | --- | --- |
| 0:00 - 0:30 | Stressor 1 – As you begin the performance, you feel sweat dripping from your hands. | 0:30  1:00  1:30  2:00  2:30 | Take a deep breath and feel confidence growing with each inhale.  Recognise that the sweating is a sign of energy and channel it into your performance.  Let the music flow and focus on each sound you create.  Remember: the audience is here to appreciate your art, not to judge you.  Use your breathing to centre yourself in the present moment. |
| 3:00 - 3:30 | Stressor 2 – You notice that your breathing is short and fast. | 3:30 –  4:00  4:30  5:00  5:30 | Inhale counting to four, exhale counting to six, bringing calm and steadiness.  Feel the air filling your body and imagine it energising your musical expression.  Use each exhale to release tension and bring clarity to the moment.  Focus on the sensation of singing/playing freely, without haste.  Visualise yourself expressing emotions with ease and tranquillity. |
| 6:00 - 6:30 | Stressor 3 – You hear a wrong note and fear making more mistakes. | 6:30  7:00  7:30  8:00  8:30 | Focus on the next sound or musical phrase, leaving the mistake behind.  Remember: mistakes are part of the process; the audience values emotional expression.  Focus on the story within the music, which goes beyond technical detail.  Imagine each sound as a piece of art that flows naturally.  Use the mistake as a reminder to connect even more deeply with the music. |
| 9:00 - 9:30 | Stressor 4 – Your vision becomes blurry for a moment as you focus. | 9:30  10:00  10:30  11:00  11:30 | Blink gently and refocus, bringing clarity back to your vision.  Take a deep breath and feel your presence on stage, anchored in the moment.  Remember: the performance is the whole, not a single detail. Focus on the bigger picture.  Use this moment to recentre yourself and reconnect with the meaning of the music.  Feel the movements of the performance flowing naturally. |
| 12:00 - 12:30 | Stressor 5 – You feel your heart beating so fast that it seems to echo through the room. | 12:30  13:00  13:30  14:00  14:30 | Feel your heartbeat slowing down as you connect with the music.  Imagine your heart as a natural metronome, adjusting itself to the moment.  Feel your inner rhythm aligning with the rhythm of the music.  Use the sound of the music to soothe yourself and calm your heartbeat.  Focus on the pleasure of being on stage, fully embracing the moment. |

## Session 2: Adaptation – Identifying Vulnerabilities and Strategies

| **Time** | **Stress induction phrase** | **Time** | **Control induction phase** |
| --- | --- | --- | --- |
| 0:00 - 0:30 | During the performance, you momentarily forget the next passage of the piece. | 0:30  1:00  1:30  2:00  2:30 | Take a deep breath and mentally visualise the next phrase or section of the music.  Reconnect with the emotional message you want to convey.  Let the melody flow naturally, just as you rehearsed.  Remember that the audience appreciates expression, not perfection.  Inspire confidence in yourself and let the music take its course. |
| 3:00 - 3:30 | You notice that you are speeding up the tempo or the musical phrase. | 3:30 –  4:00  4:30  5:00  5:30 | Adjust the tempo, feeling the flow and correct timing in your body.  Breathe and imagine each note or word aligned with the pulse of the music.  Reconnect with the natural beat of the melody or accompaniment.  Feel the rhythm stabilising as the music moves forward.  Trust your preparation and readjust without hurry or pressure. |
| 6:00 - 6:30 | Your mouth is dry and you feel difficulty swallowing. | 6:30  7:00  7:30  8:00  8:30 | Imagine taking a sip of cool water and feel your throat relax.  Take a deep breath, bringing calm and comfort to your vocal expression.  Focus on the sound you are creating instead of the physical sensations.  Use your breathing to mentally ‘rehydrate’ and relieve tension.  Visualise your voice flowing with clarity and naturalness. |
| 9:00 - 9:30 | You hear an unexpected noise in the environment that distracts you. | 9:30 –  10:00  10:30  11:00  11:30 | Focus on the music and let the noise blend into the background.  Reconnect with the sound of your voice or the musical accompaniment.  Imagine yourself in a space where only the music is the centre of attention.  Use the noise as a reminder to focus on the present moment.  Remember: the audience is with you, absorbing your music — not the noise. |
| 12:00 - 12:30 | You notice a slight tremor in your hands, lips, or voice. | 12:30  13:00  13:30  14:00  14:30 | Relax your muscles, allowing the tension to gradually dissipate.  Focus on the control and precision of your technique.  Imagine your movements or your voice as soft and expressive.  Inhale confidence and feel grounded by the music you are creating.  Remember that the tremor fades as you immerse yourself in the performance. |

## Sessão 3: Identificação dos Sintomas - Conexões entre Respiração, Neurofeedback e Sintomas

| **Time** | **Stress induction phrase** | **Time** | **Control induction phase** |
| --- | --- | --- | --- |
| 0:00 - 0:30 | You feel your chest tighten as you prepare to perform. | 0:30  1:00  1:30  2:00  2:30 | Take a deep breath, feeling your chest expand gently.  Imagine your chest opening up to let the music flow.  Relax your shoulders and feel your body aligning with your breath.  Reconnect with the positive emotion you want to express.  Remember: this is your moment, and you are prepared to make the most of it. |
| 3:00 - 3:30 | \| Your breathing becomes noisy and irregular during the performance. \| \| --- \|  \|  \| \| --- \| | 3:30 –  4:00  4:30  5:00  5:30 | Listen to your breath becoming softer and more regular.  Breathe slowly through your nose, bringing calm to your body.  Use your breath to recentre yourself in the rhythm of the music.  Imagine your voice or your sound flowing in harmony with your breathing.  Focus on the regular cadence of the music and align yourself with it. |
| 6:00 - 6:30 | You feel a sudden heat rising through your face while you are playing or singing. | 6:30  7:00  7:30  8:00  8:30 | Imagine a refreshing breeze passing across your face.  Feel the music cooling and calming your entire body.  Take a deep breath, allowing the warmth to dissipate with each exhale.  Visualise yourself performing with serenity, regardless of the sensation.  Remember: the audience is focused on your art, not on your sensations. |
| 9:00 - 9:30 | A tingling sensation appears in your hands halfway through the piece. | 9:30 –  10:00  10:30  11:00  11:30 | Move your fingers gently and regain steadiness.  Feel the flow of energy in your hands stabilising with each movement.  Focus on the lightness and precision of your gestures.  Take a deep breath, visualising your hands performing with confidence.  Recognise the sensation as a temporary moment and continue expressing yourself. |
| 12:00 - 12:30 | You become aware of your heart beating strongly during a technical passage. | 12:30  13:00  13:30  14:00  14:30 | Take a deep breath and feel your heartbeat stabilising.  Use your breathing to calm the rhythm of your heart.  Imagine your heart aligning with the rhythm of the music.  Focus on the next phrase, leaving the nervousness behind.  Remember: the technique is in your body — you only need to let it flow naturally. |

## Session 4: Identification of Symptoms – Cognitive and Behavioural Strategies

| **Time** | **Stress induction phrase** | **Time** | **Control induction phase** |
| --- | --- | --- | --- |
| 0:00 - 0:30 | Your mind fills with thoughts about failing. | 0:30  1:00  1:30  2:00  2:30 | Bring your attention to the melody and let the thoughts pass.  Take a deep breath and focus on the emotion you want to convey.  Imagine each sound as part of a story you are telling.  Concentrate on the present moment, feeling the music flow.  Remember: imagined mistakes do not define your performance. |
| 3:00 - 3:30 | You feel tension in your shoulders while you are playing or singing. | 3:30  4:00  4:30  5:00  5:30 | Consciously release your shoulders.  Take a deep breath and let the tension flow down your arms.  Visualise your shoulders light and loose, allowing freedom of movement.  Focus on the fluidity of your gestures or your voice, releasing any stiffness.  Remember that lightness enhances the quality of your musical expression. |
| 6:00 - 6:30 | Your fingers feel stiff during a fast passage. | 6:30  7:00  7:30  8:00  8:30 | Flex your fingers and feel them become agile.  Relax your hands and visualise the movements flowing naturally.  Imagine each note or gesture emerging with precision and ease.  Breathe and feel your fingers aligned with the energy of the music.  Trust your practice and let your body lead the movement. |
| 9:00 - 9:30 | A small mistake makes you think you’ve ruined everything. | 9:30 –  10:00  10:30  11:00  11:30 | Focus on the next musical phrase.  Remember: the audience rarely notices small mistakes.  Take a deep breath and use the mistake as momentum for a more expressive performance.  Concentrate on the narrative of the music, leaving the mistake behind.  Imagine the mistake dissolving as you move forward with confidence. |
| 12:00 - 12:30 | The sound of your instrument or voice comes out differently than expected. | 12:30  13:00  13:30  14:00  14:30 | Gently adjust and feel the sound aligning.  Take a deep breath and let the sound adapt to the moment.  Focus on the quality and intention of your interpretation.  Visualise the ideal sound and work with what you have in the present.  Remember: emotional expression is more important than technical perfection. |

## Session 5: Enhancement – Refining Individual Strategies

| **Time** | **Stress induction phrase** | **Time** | **Control induction phase** |
| --- | --- | --- | --- |
| 0:00 - 0:30 | You notice a slight pain in your arms while you are playing or singing. | 0:30  1:00  1:30  2:00  2:30 | Relax your arms and feel them balanced and light.  Take a deep breath and imagine your arms floating gently.  Adjust your posture to distribute the weight comfortably.  Focus on the fluidity of the movement, letting the stiffness disappear.  Feel your arms as a natural extension of your musical expression. |
| 3:00 - 3:30 | When you play or sing, you notice that your muscles are very tense. | 3:30 –  4:00  4:30  5:00  5:30 | Relax the way you hold the instrument, keeping it firm but comfortable.  Visualise your hands working in harmony with the instrument.  Focus on the sound being produced, allowing your hands to stay relaxed.  Take a deep breath and gradually release the tension in your hands and fingers.  Remember that a light touch improves control and precision. |
| 6:00 - 6:30 | Your breathing becomes fast during a challenging passage. | 6:30  7:00  7:30  8:00  8:30 | Breathe slowly, feeling each inhale bring stability.  Count to four as you inhale and to six as you exhale, regulating the rhythm.  Focus on your breathing to anchor yourself in the present moment.  Imagine your breath as a solid foundation for the performance.  Adjust your posture to distribute the weight comfortably. |
| 9:00 - 9:30 | You feel accumulated tension in your jaw. | 9:30 –  10:00  10:30  11:00  11:30 | Release your jaw and allow the tension to dissolve.  Take a deep breath and feel the relaxation spreading across your face.  Gently move your jaw to ease the stiffness.  Focus on the softness of your facial expression while you play or sing.  Remember that a relaxed face enhances your musical expression. |
| 12:00 - 12:30 | You hear your colleagues playing and fear that you may be out of sync. | 12:30  13:00  13:30  14:00  14:30 | Focus on your sound and gently adjust the tempo.  Take a deep breath and trust your place within the ensemble.  Imagine yourself flowing in harmony with the other musicians.  Feel the music as a conversation where each sound has its space.  Remember that small adjustments maintain connection and balance. |

## Session 6: Enhancement – Control of Physiological Reactions

| **Time** | **Stress induction phrase** | **Time** | **Control induction phase** |
| --- | --- | --- | --- |
| 0:00 - 0:30 | You feel increasing pressure in your forehead during the performance. | 0:30  1:00  1:30  2:00  2:30 | Relax your forehead, letting it become soft and calm.  Take a deep breath and feel the relaxation spreading across your forehead.  Imagine the tension gently melting away, leaving you feeling light.  Focus on the sound or melody, allowing the tension to fade.  Concentrate on your musical expression and feel your forehead relax naturally. |
| 3:00 - 3:30 | Your breathing seems to catch in the middle of the performance. | 3:30  4:00  4:30  5:00  5:30 | Take a deep breath and feel the air flowing gently.  Count to four as you inhale and to six as you exhale to regulate the rhythm.  Focus on your breathing as the foundation of your performance.  Visualise your breath feeding your voice or technique with energy.  Let your breathing guide you in a calm and steady way. |
| 6:00 - 6:30 | You become aware of your restless feet. | 6:30  7:00  7:30  8:00  8:30 | Press your feet firmly into the floor, feeling stability.  Feel the contact of your feet with the ground as a secure point of support.  Imagine invisible roots connecting you to the ground, bringing balance.  Take a deep breath and let the stability of your feet strengthen your confidence.  Use the gentle movement of your feet to connect yourself with the rhythm of the music. |
| 9:00 - 9:30 | A slight tremor appears in your knees while you are playing or singing. | 9:30  10:00  10:30  11:00  11:30 | Feel your knees steady as you anchor yourself in the rhythm of the music.  Relax the muscles in your legs, allowing the tremor to lessen.  Visualise your legs as stable pillars that support you.  Focus on the rhythm or the sound, allowing your body to align.  Take a deep breath, bringing calm to your knees and legs. |
| 12:00 - 12:30 | Your vision seems blurry for a moment. | 12:30  13:00  13:30  14:00  14:30 | Blink softly and visualise the sheet music clearly.  Take a deep breath and feel clarity returning with each blink.  Focus on the present moment and let your eyes adjust naturally.  Imagine the sheet music or the scene with sharpness in your mind.  Remember that small visual adjustments do not compromise the performance. |

## Session 7: Conditioning – Practising Control Strategies

| **Time** | **Stress induction phrase** | **Time** | **Control induction phase** |
| --- | --- | --- | --- |
| 0:00 - 0:30 | Your mind becomes distracted for a moment during the performance. | 0:30  1:00  1:30  2:00  2:30 | Bring your attention back to the music, one note at a time.  Take a deep breath and use each sound to reconnect with the present.  Focus on the emotion you want to convey through the music.  Visualise the next phrase or passage, letting the distraction fade away.  Remember that returning to focus is a natural skill and fully within your reach. |
| 3:00 - 3:30 | You notice that the sound of the instrument seems weaker. | 3:30  4:00  4:30  5:00  5:30 | Adjust the pressure on the instrument, feeling the sound return to balance.  Breathe and trust your technical control to restore the sound.  Visualise the ideal sound and work gently toward achieving it.  Focus on the consistency of the sound rather than its momentary intensity.  Remember that gradual adjustments create significant improvements. |
| 6:00 - 6:30 | You feel your hands becoming cold halfway through the piece. | 6:30  7:00  7:30  8:00  8:30 | Imagine a gentle warmth spreading through your hands.  Move your fingers lightly, feeling the warmth and energy flowing.  Take a deep breath and visualise your body warming up naturally.  Focus on the lightness and precision of your hand movements.  Remember that your emotional connection to the music warms your expression. |
| 9:00 - 9:30 | An unexpected note sounds wrong. | 9:30  10:00  10:30  11:00  11:30 | Let the wrong note pass and focus on the next one with confidence.  Remember that the audience focuses on the whole, not on the mistake.  Take a deep breath and use the next note to restore harmony.  Focus on the feeling and intention of the music, not on the details.  Imagine the mistake dissolving like a cloud in the clear sky of the music. |
| 12:00 - 12:30 | You notice a sudden tension in your neck. | 12:30  13:00  13:30  14:00  14:30 | Relax your neck and feel lightness returning.  Take a deep breath and feel the muscles in your neck releasing.  Adjust your posture to bring more comfort and freedom.  Imagine your neck as a light yet stable support for your performance.  Focus on the fluidity of your movements and the serenity of your expression. |

## Session 8: Simulation and Monitoring Exercise

| **Time** | **Stress induction phrase** | **Time** | **Control induction phase** |
| --- | --- | --- | --- |
| 0:00 - 0:30 | Your breathing begins to become short and irregular. | 0:30  1:00  1:30  2:00  2:30 | Breathe slowly and deeply, bringing regularity and calm.  Count to four as you inhale and to six as you exhale, stabilising the rhythm.  Imagine each breath feeding your performance with energy.  Focus on the natural movement of the diaphragm to regulate your breathing.  Let the music guide the rhythm of your breath. |
| 3:00 - 3:30 | You feel a tightness in the muscles of your back. | 3:30  4:00  4:30  5:00  5:30 | Relax the muscles in your back, letting them loosen and become comfortable.  Adjust your posture to relieve tension in your back.  Take a deep breath, visualising the tension disappearing with each exhale.  Imagine your back as a flexible yet stable support.  Focus on the comfort of your body as you connect with the music. |
| 6:00 - 6:30 | Your fingers feel stiff and slow. | 6:30  7:00  7:30  8:00  8:30 | Gently flex your fingers and feel your agility returning.  Move your fingers lightly, feeling the energy flowing naturally.  Visualise your fingers moving with fluidity and precision.  Take a deep breath and feel your fingers responding to your command.  Trust your practice and let the movements happen effortlessly. |
| 9:00 - 9:30 | The light seems too bright and distracts your vision. | 9:30  10:00  10:30  11:00  11:30 | Focus on the sheet music, adjusting your vision to the ambient light.  Blink softly, allowing your eyes to adapt to the lighting.  Take a deep breath and concentrate on the details of the sheet music or the stage.  Imagine the light as part of the scenery that enhances your music.  Use the sound to strengthen your connection with the performance, ignoring the light. |
| 12:00 - 12:30 | You hear an external sound that breaks your concentration. | 12:30  13:00  13:30  14:00  14:30 | Ignore the external sound and bring your focus back to your performance.  Concentrate on the sound you are creating as the centre of your attention.  Take a deep breath and use your music to neutralise the distraction.  Imagine yourself in a space where only the sound of your performance matters.  Remember that the audience is focused on your music, not on external noises. |

**
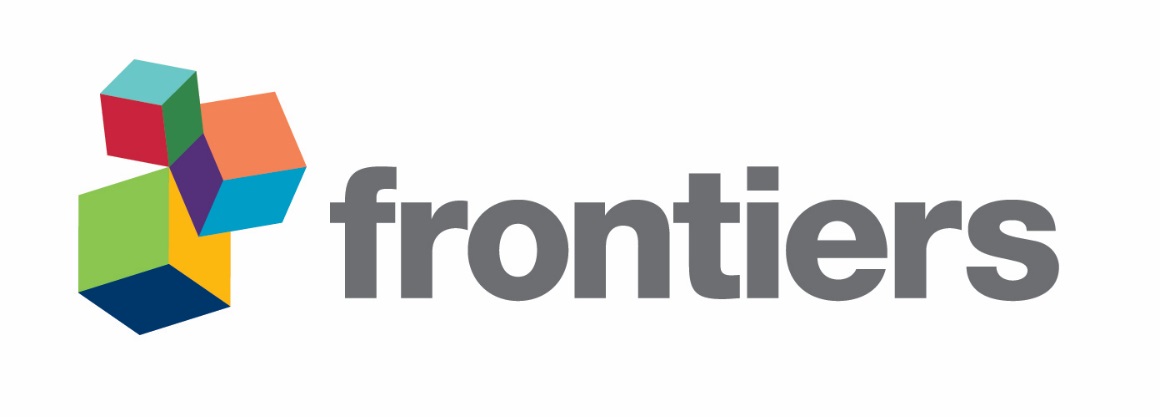
**
